# Promoting occupational justice policies in mental health organizations: A model based on the experiences of mental health rehabilitation consumers and employees

**DOI:** 10.1192/j.eurpsy.2023.1141

**Published:** 2023-07-19

**Authors:** H. Cohen, N. Schreuer, D. R. Vashdi

**Affiliations:** University of Haifa, Haifa, Israel

## Abstract

**Introduction:**

: Occupational justice (OJ) regards the human right to be engaged in meaningful life occupations (work, leisure, learning, house management etc.). It highlights the idea that all society members should be able to actively participate in all occupations as equals. Yet, people with mental health problems remain at the margins of society and struggle to fully participate in life activities. At the same time, such participation has been shown to lead to better functional abilities, higher quality of life, and better illness management among this population. It provides routine, connectedness, belonging, purpose, and identity. Moreover, impaired occupational participation due to mental health problems has resulted in functional impairment, symptomatic deterioration, loss of social roles, and a reduced sense of competence. Despite the importance of such participation, it is unclear in many mental health rehabilitation service organizations how to design policies that will achieve better occupational participation for their consumers.

**Objectives:**

To better understand the OJ concept and to create a conceptual model pertaining to the challenges and solutions, which may serve policy makers as a theoretical basis for enhancing OJ based policies.

**Methods:**

We conducted a qualitative phenomenological study that included in-depth interviews with mental health rehabilitation consumers, to identify their experiences and perceptions in relation to OJ and focus groups with rehabilitation employees and managers. Data analysis was a multi-staged process using a systematic and inductive procedure.

**Results:**

Based on our analysis, the three key themes clarifying the interviewees’ experiences with the OJ concept were a) the importance of and barriers for achieving meaningful participation, b) the required resources for implementing OJ, and c) principles for practice. These elements comprise a holistic OJ construct, affording a practical understanding of what a service that implements OJ means. Such a service would use the resources and practices discussed in this study and address meaningful participation as the desired outcome to achieve.

**Conclusions:**

Unlike other forms of justice, OJ emphasizes the need for flexible and tailor-made services that address the consumer’s changing needs and circumstances. It considers the consumers’ role as active rather than passive service recipients. Recently, the fields of health and rehabilitation have increasingly acknowledged the importance and applications of involving consumers. Their genuine involvement would enhance OJ and provide a basis for more accurate assessments and customized interventions.

**Disclosure of Interest:**

None Declared

